# Information Prioritization Underpins the Flexible Expression of Social Preferences Under Time Constraints

**DOI:** 10.1177/19485506251314071

**Published:** 2025-01-28

**Authors:** Yi Yang Teoh, Hyuna Cho, Cendri A. Hutcherson

**Affiliations:** 1 University of Toronto, Ontario, Canada

**Keywords:** attention, individual differences, information search, altruism, cooperation, time pressure, social preferences

## Abstract

While recent research shows how time constraints exacerbate the influence of contextual (dis)incentives on information prioritization and subsequent choice during prosocial decision-making, this emerging perspective is silent on how pervasive individual differences in dispositional social preferences might interact with these contextual factors to shape these processes. To bridge this gap, we demonstrated in a preregistered study (*N* = 200 adults from the United States and Canada; Prolific Academic) that people calibrate their information priorities based on both their dispositional social preferences and contextual (dis)incentives, and that time constraints further exacerbated information prioritization that aligned with their own social preferences, in addition to information incentivized by the broader social context. Furthermore, these information priorities subsequently biased prosocial choices, extremifying people’s selfish/prosocial choice patterns under time constraints. These findings suggest that flexible information prioritization underpins people’s capacity to navigate different social interactions while balancing their own preferences against external incentives and constraints.

## Introduction

People’s capacity to care for other individuals and groups, even at the expense of their own immediate interests, forms the bedrock of human societies. While research has found that people sometimes make sacrifices for others’ welfare, these prosocial tendencies vary widely across individuals and contexts (Bester & Güth, 1998; Fehr & Fischbacher, 2004; Hein et al., 2016; Henrich et al., 2005; Piliavin & Charng, 1990). Recent evidence suggests that these varying patterns of selfish and prosocial behavior might be explained by differences in attention allocation and information search across situational contexts (Teoh et al., 2020). This work theorizes that *social decision-making is constrained by processes of information search.* When faced with a choice to be prosocial or selfish, people must first search for relevant information about their own and others’ outcomes before they can evaluate each option. Popular experimental manipulations of prosocial behavior such as time constraints can thus make people choose more selfishly or generously simply by limiting the amount of information people search for—that is, making people more selfish by preventing them from learning how their choices impact others.

Importantly, complementing this theory, research shows that people flexibly adapt to these constraints by prioritizing contextually relevant information during search, but that this then systematically biases the social decisions they make (Teoh & Hutcherson, 2022). When there are no repercussions for selfish behavior such as when playing a Dictator game, people increasingly prioritize self-relevant information, ignore other-relevant information, and make more selfish decisions under time constraints. In contrast, when selfish behavior could be penalized in an Ultimatum game, people tend to prioritize self-relevant information less and other-relevant information more under time constraints, leading to more prosocial choices overall. However, although this theoretical framework provides new insights into how context shapes people’s social behavior through attention, relevant research has not yet considered *whether* and *how* individual differences in people’s dispositional social preferences interact with these contextual factors. Critically, such work could shed new insight into fundamental socio-cognitive processes that enable people to flexibly adapt to a myriad of social situations and inform more effective, personalized interventions to increase prosocial behavior.

In particular, an extensive body of work has documented robust individual differences in whether time constraints make people more or less prosocial (Chen & Krajbich, 2018; Chen, Zhu, et al., 2024; Cornelissen et al., 2011; Pancotto & Righi, 2021). Time pressure tends to exacerbate individual differences in social preferences: selfish people make more selfish choices under time constraints while prosocial people make more prosocial choices. Often, researchers interpret these findings as evidence for dual-process theories, reasoning that time pressure constrains deliberation and biases behavior toward people’s idiosyncratic social intuitions. However, here, we extend the aforementioned attentional framework and propose instead that individual differences in time pressure’s effects on prosociality result parsimoniously from flexible information prioritization. Because selfish individuals fundamentally care more about their own interests and prosocial individuals care more about others, we predict that selfishly predisposed individuals ought to prioritize self-relevant information, while prosocial individuals ought to prioritize other-relevant information, especially under time constraints. Since time constraints might force people to choose without sampling sufficient information, information priorities could then bias their choices more strongly, leading to the extremification of peoples’ overall pattern of selfish/prosocial decisions.

A key advantage of our theory of information prioritization is that it provides a parsimonious and unified account of both individual and contextual differences in time pressure’s effects on prosociality. Although a simple investigation showing that dispositional social preferences shape information priorities and choice under time constraints would provide evidence of this, it would fall short of capturing how social interactions in the real world often require people to *simultaneously* contend with external (dis)incentives that conflict with their dispositional preferences on top of constraints on decision-making like time.

Thus, in this article, we seek to demonstrate that people flexibly adapt their information search to *jointly* prioritize both their own dispositional social preferences *and* any (dis)incentives in the social environment to optimize decision-making under constraints. In doing so, we first aim to replicate existing findings that time pressure exacerbates both contextual and individual differences in prosocial choices. Second, we test our predictions that time pressure drives people to prioritize information search more strongly for both contextually relevant—that is, (dis)incentive-compatible—information *and* information that aligns with their social preferences. Here, we specifically consider how potential punishment from others in the Ultimatum Game, but not the Dictator Game, powerfully disincentivizes the free prioritization of information search based on personal social preferences. Finally, and most importantly, we expect that changes in people’s prosocial behavior under time constraints are fully explained by information prioritization during decision-making.

## Method

### Overview

To test our predictions, we conducted a preregistered experiment that measured participants’ information search patterns and prosocial decisions in Dictator and Ultimatum games under different levels of time pressures. We recruited a gender-balanced sample of participants from the U.S. and Canada through Prolific Academic (final *N* = 200; gender: 102 men, 95 women, 2 nonbinary, 1 preferred not to answer; Age: *M* [*SD*] = 31.1 [9.60], Min = 18, Max = 67). To quantify individual differences in participants’ dispositional social preferences, we first measured their Social Value Orientation (SVO) using the primary six-item measure (Murphy et al., 2011) before they were randomly assigned to play either the Dictator (*N* = 100) or Ultimatum Game (*N* = 100). We omitted the SVO’s secondary items which aim to differentiate between specific aversions to inequity that are not immediately relevant to our current investigation. We clarify that we *do not* assume that SVO is a pure metric of social preference independent of and separate from measures of such preferences within the games. Instead, we specifically leverage the fact that all these measures tap into similar underlying processes and use SVO as a common metric of individual differences across the games to more precisely disentangle effects of individual and contextual differences on information search and choice.

Our sample (SVO: *M* [*SD*] = 27.9 [13.0], Min = 7.82, Max = 46.0) consisted of 141 prosocial people who valued others’ welfare but to a lesser extent than their own, 22.45 < SVO < 57.15, and 59 individualists who valued others’ welfare minimally, −12.04 < SVO < 22.45. Unsurprisingly, our sample had no hyper-altruists (those who valued others’ welfare more than their own, SVO > 57.15). Average SVO did not differ between our conditions (*b* = 2.703, 95% confidence interval [CI] = [−0.902, 6.309], *t*(198) = 1.478, preregistered two-tailed *p* = .141, *r* = 0.104). We preregistered our sample size of *N* = 200, with *N* = 100 for each condition based on bootstrapped power analyses that estimated a power of 0.857 for the two-way interaction between time pressure and game context. A post hoc conservative estimation suggests that our sample size would yield a simultaneous power of 0.857^2^ = 0.734 for two independent sets of analogous effects (i.e., game context × time pressure, SVO × time pressure).

Our study deployed versions of the Dictator and Ultimatum games previously used to simultaneously track participants information search during social decision-making (Teoh & Hutcherson, 2022). The experiment comprised a series of trials where we presented participants with a proposed distribution of money for themselves and another person. Participants were then asked to decide whether they would like to accept or reject the proposed distribution over a default split of $50 each. Participants in the Dictator game were then told that their choices would fully determine theirs and their partners’ outcomes: accepting would result in the proposed distribution for them and their partner, while rejecting would result in $50 for both them and their partner. In contrast, in the Ultimatum game, participants were told that their choices to accept or reject the proposed distribution over the default would additionally be subject to their partners’ approval. If participants’ partners accepted the decision, the choice would be implemented. If participants’ partners did not approve of the participants’ choices, the choices would be vetoed and neither party would receive any money. However, to eliminate potential learning effects, participants were only informed about their partners’ approval on a randomly selected trial at the end of the experiment, which determined the monetary bonus for both them and their partners.

**Figure 1.**
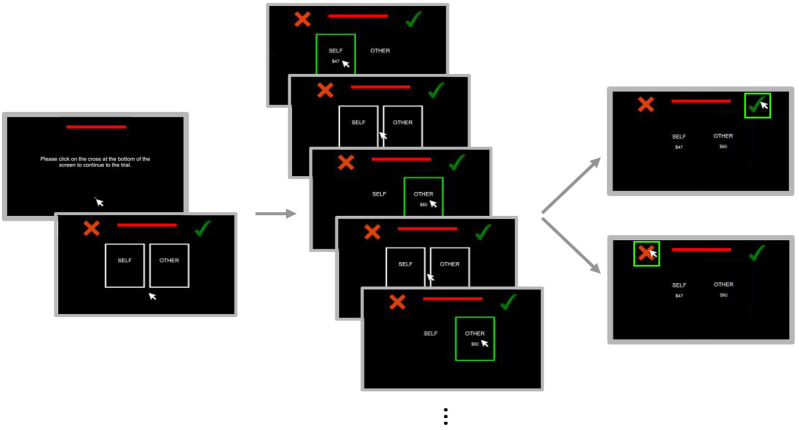
Graphical Schematic of a Trial in the Decision-Making Task Participants begin each trial by using the mouse to click on a cross in the center of the screen. They can then sample information by hovering their mouse cursor over the self or other areas-of-interest or make a choice by clicking on either the check mark (accept the proposal) or cross (choose the default). The length of a horizontal bar at the top of the screen signaled whether they were in the low (long bar) or high (short bar) time pressure condition.

During the experimental task, we measured participants’ information search using mouse-tracking in addition to participants’ choices and response times (see Figure 1). Specifically, monetary amounts for the participant and their partner in the proposed distribution were not immediately available upon trial onset. Instead, participants began each trial by clicking on a central cross that re-centered their mouse position. They could then move their mouse cursor over one of two spatially defined areas-of-interest (AOIs) to reveal outcomes for either themselves ($Self) or their partner ($Other), which remained visible onscreen until they moved their cursor outside the AOI. Participants were told to sample as much information as they wanted from the AOIs before accepting (clicking a green check mark) or rejecting (clicking a red cross) the proposed distribution. Critically, the left-right mapping of AOIs and response buttons were fully counterbalanced across participants, but consistent trial-to-trial.

To manipulate time pressure, participants’ information search and choices were constrained by either a time limit of 1.5 seconds (high time pressure) or 10 seconds (low time pressure). This was visually indicated by the length of a red bar at the top of the screen. In addition, participants were prohibited from making choices within the first 1.5 seconds of trials in the low time pressure condition, although they were permitted to sample information during this time. Participants completed practice blocks of 22 trials under low then high time pressure before completing 10 experimental blocks of 22 trials where time pressure varied pseudo-randomly from block to block. Participants never encountered more than two consecutive blocks with the same time pressure condition.

The amounts of money proposed for the participant and their partner on a given trial each varied from $ 0 to $100. To calculate participants’ bonuses, the outcomes of a randomly selected trial were divided by a factor of 50 into U.S. dollars. Participants were informed that their bonus would scale with the amounts in the experiment but remained ignorant of the precise exchange rate until the end of the experiment. Participants were additionally compensated 5 U.S. dollars for their time. Informed consent was collected based on a protocol approved by the Research Ethics Board at the University of Toronto.

### Experimental Stimuli

Proposed distributions comprised of 20 unique combinations of $Self and $Other. These combinations were selected such that if $Self was greater than 50, $Other would be less than or equal to 50, or vice versa. Each combination was repeated five times in the high and low time pressure conditions (100 trials each), but modified by random variation, *U*(−2,2), to prevent the caching of responses. We added another 20 catch trials to orthogonalize the values of $Self and $Other, where both amounts were equally larger or smaller than 50. Since the experiment was delivered online to participants’ own devices using Inquisit Web 5.0.14.0, we defined the size of all stimuli relative to the device’s size but minimized variability in screen-size by requiring participants to complete the task on a desktop or laptop computer.

### Behavioral Indices

We discarded all information samples that were shorter than 100 ms in duration from analyses as preregistered, based on prior work on information processing latencies (Manor & Gordon, 2003). After removing catch trials, we defined participants’ choices as prosocial (1) when they either accepted a proposed distribution where their outcomes were smaller than the default $50, and their partners’ outcomes were greater than the $50, or (2) when they rejected a proposed distribution where their outcomes were larger than the default $50, and their partners’ outcomes were smaller than the $50. We defined all other responses as selfish. Finally, we also removed from analyses of prosociality trials those cases where participants failed to respond within the time limit of the condition (high time pressure: *M* = 6.690%; low time pressure: *M* = 0.082%) and trials where participants chose without sampling any information (i.e., guesses: high time pressure: *M* = 2.570%; low time pressure: *M* = 0.118%).

### Exclusions

Based on preregistered criteria, we removed 239 out of 439 recruited participants. Six did not complete the task, one revoked consent for data use, 35 failed comprehension checks, 61 provided the same response in more than 90% of trials, 11 failed to respond in time on more than 25% of trials in at least one of the time pressure conditions, 74 failed to make the rational response in catch trials, and 51 made choices without sampling any information in more than 50% of trials in at least one time pressure condition.

### Statistics

General linear mixed-effects regressions were conducted in R 4.3.3 (R Core Team, 2017) using the lme4 package v1.1-35.2 with degrees of freedom estimated using the Satterthwaite method (Bates et al., 2015; Kunzetsova et al., 2017). We then calculated effect sizes (*r*) through transformations of *t*-statistics in the case of linear models and transformations of odds ratios in the case of logistic models (Borenstein et al., 2011; Edwards et al., 2008; Kashdan & Steger, 2014). Model comparison and selection were performed using the Bayesian Information Criterion (BIC; Burnham & Anderson, 2004; Raftery, 1996).

### Transparency and Openness

All data, analysis code, and research materials are available at https://osf.io/yhzxd/. This study’s design and its analysis were preregistered at https://osf.io/h3dj9/. All preregistered analyses are reported in full in Supplementary Table S1 to S4.

### Time Pressure Manipulation Check

As expected, participants responded more quickly under high time pressure in both the Dictator (DG: *M* = 1,074 ms, *SD* = 119 ms) and Ultimatum game (UG: *M* = 1,095 ms, *SD* = 125 ms) than under low time pressure (DG: *M* = 2,197 ms, *SD* = 334 ms, *b_time_* = −0.707, 95% CI = [−0.712, −0.701], *t*(43796) = −267.098, two-tailed *p* < .001, *r* = 0.787; UG: *M* = 2,184 ms; *SD* = 321 ms, *b_time_* = −0.683, 95% CI = [−0.688, −0.678], *t*(43796) = −258.203, two-tailed *p* < .001, *r* = 0.777). Furthermore, time pressure constrained information search as expected (see Supplementary Table S1). Participants collected fewer information samples under high (DG: *M* = 1.39, *SD* = 0.433; UG: *M* = 1.66, *SD* = 0.418) compared to low time pressure (DG: *M* = 2.17, *SD* = 0.340, *b_time_* = −0.536, 95% CI = [−0.549, −0.523], *z* = −81.202, preregistered one-tailed *p* < .001, Incidence Rate Ratio = 0.585; UG: *M* = 2.21, *SD* = 0.366, *b_time_* = −0.382, 95% CI = [−0.393, −0.370], *z* = −65.660, preregistered one-tailed *p* < .001, Incidence Rate Ratio = 0.683). Participants were also less likely to sample both unique pieces of information within a trial under high (DG: *M* = 0.604, *SD* = 0.400; UG: *M* = 0.331, *SD* = 0.377) compared to low time pressure (DG: *M* = 0.062, *SD* = 0.105, *b_time_* = 4.483, 95% CI = [4.305, 4.662], *z* = 49.251, preregistered one-tailed *p* < .001, *r* = 0.777; UG: *M* = 0.028, *SD* = 0.057, *b_time_* = 3.917, 95% CI = [3.747, 4.086], *z* = 45.345, preregistered one-tailed *p* < .001, *r* = 0.734).

## Results

### Time Pressure Exacerbates the Influence of Both Individual Differences and Contextual Incentives on Prosocial Choice

**Figure 2.**
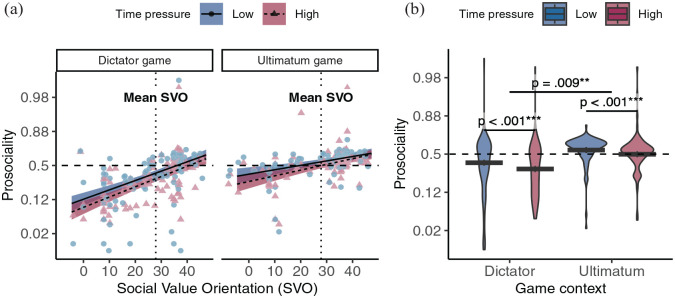
(A) Prosociality as a Function of Participants’ Social Value Orientation (SVO), Game Context, and Time Pressure. (B) Group Prosociality as a Function of Game Context and Time Pressure In (A), each point represents the proportion of prosocial choices a single participant made, shaped and colored by time pressure condition. The lines and shaded regions represent the model-predicted mean and 95% confidence intervals, colored by time pressure condition. The horizontal dashed line indicates the level of prosociality where people were just as likely to make prosocial and selfish choices. The vertical dotted line indicates the mean SVO of the sample. In (b), box plots represent the model-predicted proportion of prosocial choices for a subject with SVO equal to the sample mean. The central line indicates the mean, the upper and lower boundaries indicate one within-subjects standard error above and below the mean, and the whiskers indicate the within-subjects 95% confidence interval. Violin plots illustrate the distribution of participants’ average prosociality in each condition. Intervals between ticks on Y-axes are scaled based on the logit-transformation to mirror the analytical approach. All statistical tests were preregistered one-tailed tests unless otherwise indicated.

First, we sought to replicate existing work showing that time pressure exacerbated the influence of *both* individual and contextual factors on prosocial choice (see Figure 2 and Supplementary Table S2 for details of the full model). As predicted, we found that time pressure amplified the association between SVO and prosocial choice in both the Dictator (DG: *b_svo:time_* = 0.069, 95% CI = [−0.004, 0.143], *z* = 1.840, preregistered one-tailed *p* = .033, *r* = 0.019) and Ultimatum game (UG: *b_svo:time_* = 0.099, 95% CI = [0.038, 0.161], *z* = 3.163, preregistered one-tailed *p* < .001, *r* = 0.027), while also simultaneously exacerbating the difference in prosociality between the DG and UG (*b_game:time_* = 0.108, 95% CI = [0.018, 0.197], *z* = 2.356, preregistered one-tailed *p* = .009, *r* = 0.030).

In other words, while people with higher SVO (i.e., more prosocial preferences) made more prosocial choices compared to those with lower SVO (i.e., more individualistic preferences) even under low time pressure in both the DG (*b_svo_* = 0.713, 95% CI = [0.544, 0.881], *z* = 8.290, preregistered one-tailed *p* < .001, *r* = 0.193) and UG (*b_svo_* = 0.336, 95% CI = [ 0.175, 0.497], *z* = 4.088, preregistered one-tailed *p* < .001, *r* = 0.092), the association between SVO and prosociality became stronger under time pressure (DG: *b_svo_* = 0.782, 95% CI = [0.611, 0.952], *z* = 8.994, preregistered one-tailed *p* < .001, *r* = 0.211; UG: *b_svo_* = 0.436, 95% CI = [0.273, 0.598], *z* = 5.256, preregistered one-tailed *p* < .001, *r* = 0.119). Likewise, while participants made more prosocial choices in the UG compared to the DG even under low time pressure (*b_game_* = 0.662, 95% CI = [0.432, 0.893], *z* = 5.629, preregistered one-tailed *p* < .001, *r* = 0.180), high time pressure increased this difference in overall prosociality between participants in the UG versus DG (*b_game_* = 0.770, 95% CI = [0.538, 1.002], *z* = 6.496, preregistered one-tailed *p* < .001, *r* = 0.208). Time pressure reduced prosociality in the DG (*b_time_* = −0.326, 95% CI = [−0.392, −0.260], *z* = −9.703, preregistered one-tailed *p* < .001, *r* = −0.090) and the UG (*b_time_* = −0.218, 95% CI = [−0.279, −0.158], *z* = −7.052, preregistered one-tailed *p* < .001, *r* = −0.060), but to a lesser extent in the latter.

Given these results, we unsurprisingly also found that individual differences in SVO were less strongly associated with prosocial choices in the UG compared to the DG (under low time pressure *b_svo:game_* = −0.377, 95% CI = [−0.609, −0.144], *z* = −3.167, preregistered one-tailed *p* < .001, *r* = −0.103). However, contrary to preregistered predictions, time pressure’s exacerbation of individual and contextual differences in prosocial choices appeared independent, and the UG did not differently constrain SVO’s influence on prosociality under time pressure (*b_svo:game_* = −0.346, 95% CI = [−0.581, −0.111], *z* = −2.889, post hoc two-tailed *p* = .004, *r* = −0.095; *b_svo:game:time_* = 0.030, 95% CI = [−0.066, 0.126], *z* = 0.621, preregistered one-tailed *p* = 1, post hoc two-tailed *p* = .535, *r* = 0.008). As a caveat, the insignificant three-way interaction may have resulted from a lack of statistical power. Still, these analyses demonstrate that time pressure’s exacerbation of individual differences in prosocial choices across games remain robust even after accounting for the possibility that the UG might weaken this effect. Separately, we note here that even our most prosocial participants in the UG became less prosocial under time pressure (+1*SD* SVO *b_time_* = −0.119, 95% CI = [−0.199, −0.039], *z* = −2.921, post hoc two-tailed *p* = .003, *r* = −0.033) and return to this point later in the discussion.

### Individual Difference in Social Preferences and Contextual Incentives Jointly Determine Information Priorities Under Time Pressure

**Figure 3.**
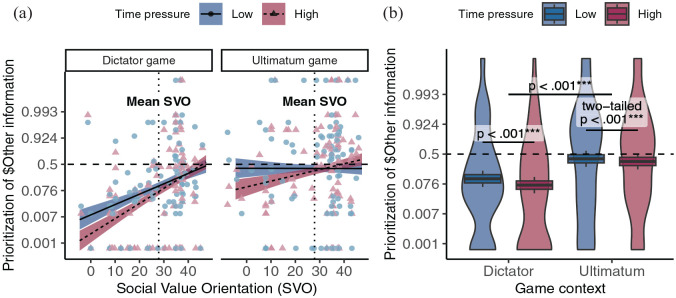
(A) Prioritization of Information About Others’ Outcomes as a Function of Participants’ Social Value Orientation (SVO), Game Context, and Time Pressure. (B) Group-Level Information Prioritization as a Function of Game Context and Time Pressure In (A), each point represents the proportion of trials a single participant searched for their partners’ outcomes first, shaped and colored by time pressure condition. The lines and shaded regions represent the model-predicted mean and 95% confidence intervals, colored by time pressure condition. The horizontal dashed line indicates the level of information prioritization where people were just as likely to search first for their partner’s outcomes as their own. The vertical dotted line indicates the mean SVO of the sample. In (B), box plots represent the model-predicted probability of prioritizing information about others for a subject with SVO equal to the sample mean. The central line indicates the mean, the upper and lower boundaries indicate one within-subjects standard error above and below the mean, and the whiskers indicate the within-subjects 95% confidence interval. Violin plots illustrate the distribution of participants’ information priorities in each condition. Intervals between ticks on Y-axes are scaled based on the logit-transformation to mirror the analytical approach. All statistical tests were preregistered one-tailed tests unless otherwise indicated.

More importantly, our theory assumes that these individual and contextual differences in prosocial choices under time pressure are driven by decision-makers’ prioritization of information to cope with processing constraints. Thus, we next sought to verify that both individuals’ SVO and the game context jointly determined what information they sampled first under these constraints (see Figure 3 and Supplementary Table S3). Our results confirmed that participants prioritized information search under time pressure according to their SVO (DG: *b_svo:time_* = 0.591, 95% CI = [0.456, 0.726], *z* = 8.559, preregistered one-tailed *p* < .001, *r* = 0.161; UG: *b_svo:time_* = 0.722, 95% CI = [0.635, 0.809], *z* = 16.316, preregistered one-tailed *p* < .001, *r* = 0.195) and the specific game context (*b_game:time_* = 0.305, 95% CI = [0.169, 0.441], *z* = 4.401, preregistered one-tailed *p* < .001, *r* = 0.084).

In the DG, selfishly predisposed participants (low SVO) more frequently searched for information about their own outcomes first compared to prosocially predisposed individuals (high SVO) even under low time pressure (*b_svo_* = 1.281, 95% CI = [0.593, 1.969], *z* = 3.649, post hoc two-tailed *p* < .001, *r* = 0.333). This bias was magnified under high time pressure (*b_svo_* = 1.872, 95% CI = [1.180, 2.563], *z* = 5.306, post hoc two-tailed *p* < .001, *r* = 0.459). Similarly, although the UG attenuated SVO’s association with information prioritization under low time pressure (*b_svo_* = −0.007, 95% CI = [−0.661, 0.648], *z* = −0.020, post hoc two-tailed *p* = .984, *r* = −0.002; *b_svo:game_* = −1.287, 95% CI = [−2.228, −0.347], *z* = −2.682, post hoc two-tailed *p* = .007, *r* = −0.334), high time pressure drove even participants in the UG to prioritize information search based on their SVO (*b_svo_* = 0.716, 95% CI = [0.053, 1.379], *z* = 2.116, post hoc two-tailed *p* = .034, *r* = 0.194).

Simultaneously, as expected, participants in the UG as a whole also tended to prioritize gathering information about their partners’ outcomes compared to those in the DG, even under low time pressure (*b_game_* = 1.673, 95% CI = [0.725, 2.621], *z* = 3.458, preregistered one-tailed *p* < .001, *r* = 0.419), and this difference in information priorities grew under high time pressure (*b_game_* = 1.978, 95% CI = [1.036, 2.920], *z* = 4.114, preregistered one-tailed *p* < .001, *r* = 0.479). Time pressure tended to drive people to more strongly prioritize information about their own outcomes in the DG (*b_time_* = −0.526, 95% CI = [−0.629, −0.423], *z* = −10.012, preregistered one-tailed *p* < .001, *r* = −0.144). Unexpectedly, time pressure also drove people to more strongly prioritize information about their own outcomes in the UG (*b_time_* = −0.221, 95% CI = [−0.310, −0.132], *z* = −4.883, preregistered one-tailed *p* = 1, post hoc two-tailed *p* < .001, *r* = −0.061), although to a lesser extent than the DG. While this result appears to contradict predictions that the UG ought to increase the relevance of others’ outcomes, these overall patterns of self-prioritization in UG are consistent with a confluence of time pressure’s interaction with individual differences in prosocial dispositions and contextual (dis)incentives. We further elaborate on this point in the discussion. Importantly, similar to choice patterns, time pressure exacerbated individual differences in information search independent of differences in game context (three-way *b_svo:game:time_* = 0.131, 95% CI = [−0.029, 0.292], *z* = 1.601, preregistered one-tailed *p* = 1, post hoc two-tailed *p* = .109, *r* = 0.036; under high time pressure *b_svo:game_* = −1.156, 95% CI = [−2.088, −0.224], *z* = −2.431, post hoc two-tailed *p* = .015, *r* = −0.304).

### Information Priorities Strongly Drive Prosocial Choice Under Time Pressure

**Figure 4.**
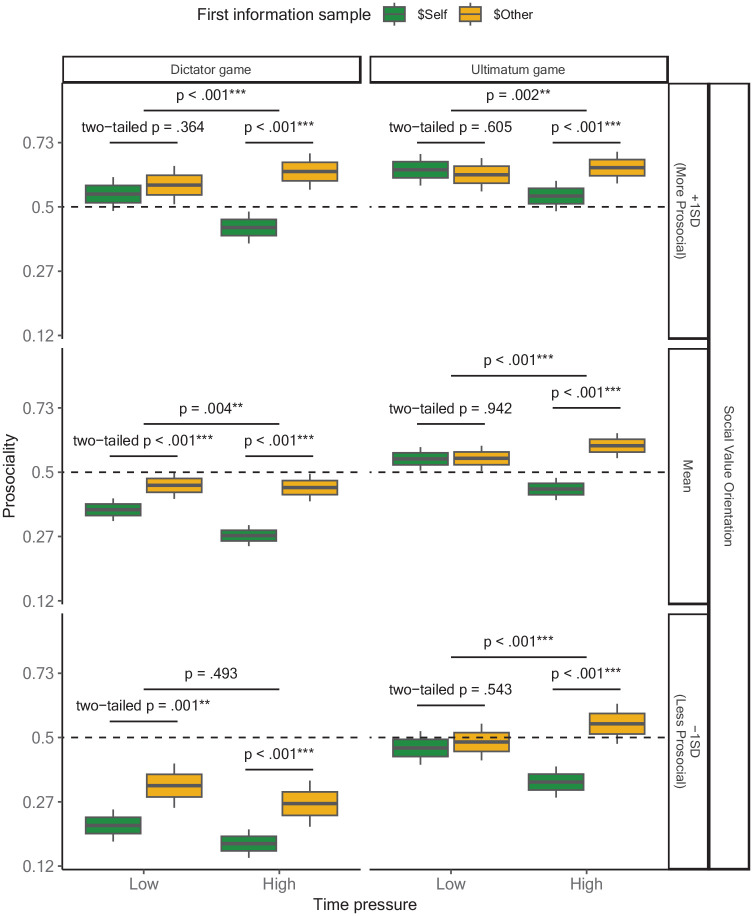
Trial-Level Effects of First Information Sample on Prosociality as a Function of Participants’ Social Value Orientation (SVO), Game Context, and Time Pressure Box plots represent the model-predicted probability of a prosocial choice. The central line indicates the mean, the upper and lower boundaries indicate one within-subjects standard error above and below the mean, and the whiskers indicate the within-subjects 95% confidence interval. The horizontal dashed line indicates the level of prosociality where people were just as likely to make prosocial and selfish choices. Intervals between ticks on Y-axes are scaled based on the logit-transformation to mirror the analytical approach. All statistical tests were preregistered one-tailed tests unless otherwise indicated. Two-tailed tests conducted were post hoc comparisons.

While we have shown thus far that time pressure interacts with individual and contextual differences to produce similar patterns of information prioritization and behavior change in social decision-making, our theory makes the strong prediction that it is precisely this strategic prioritization of information that underlies time pressure’s exacerbation of individual and contextual differences in prosocial choices. Specifically, because time pressure constrains opportunities to gather information, any information acquired first should have a much larger effect on prosociality under time pressure than it does under free response. This should be true regardless of individual differences in social preferences and game context. As expected, using a mixed-effects logistic model predicting trial-by-trial prosocial choices from first information samples, SVO, game context, time pressure, and their interactions (see Figure 4 and Supplementary Table S4), we found that time pressure interacted with first information samples to predict prosocial choices on each trial in both the DG and UG for most participants: whether they held moderate prosocial preferences (at mean SVO in the DG *b_info1:time_* = 0.367, 95% CI = [0.099, 0.636], *z* = 2.684, preregistered one-tailed *p* = .004, *r* = 0.101; UG *b_info1:time_* = 0.666, 95% CI = [0.389, 0.943], *z* = 4.717, preregistered one-tailed *p* < .001, *r* = 0.181; three-way *b_info1:time:game_* = 0.299, 95% CI = [−0.092, 0.689], *z* = 1.500, post hoc two-tailed *p* = .134, *r* = 0.082), or strong prosocial preferences (at +1*SD* SVO in the DG *b_info1:time_* = 0.731, 95% CI = [0.357, 1.105], *z* = 3.829, preregistered one-tailed *p* < .001, *r* = 0.198; UG *b_info1_*_:__*time*_ = 0.518, 95% CI = [0.157, 0.879], *z* = 2.815, preregistered one-tailed *p* = .002, *r* = 0.141; three-way *b_info1:time:game_* = −0.213, 95% CI = [−0.717, 0.291], *z* = −0.829, post hoc two-tailed *p* = .407, *r* = −0.059). However, in this full model, we also found that for participants with weak prosocial preferences, first information samples were only more predictive of prosocial choice under high time pressure in the UG (at −1*SD* SVO *b_info1:time_* = 0.815, 95% CI = [0.423, 1.206], *z* = 4.073, preregistered one-tailed *p* < .001, *r* = 0.219), and not in the DG (*b_info1:time_* = 0.004, 95% CI = [−0.444, 0.452], *z* = 0.017, preregistered one-tailed *p* = .493, *r* = 0.001; three-way *b_info1:time:game_* = 0.811, 95% CI = [0.213, 1.408], *z* = 2.661, post hoc two-tailed *p* = .008, *r* = 0.218; four-way *b_info1:time:game:svo_* = −0.512, 95% CI = [−0.927, −0.097], *z* = −2.420, post hoc two-tailed *p* = .016, *r* = −0.140). Post hoc analyses suggest, however, that this is because, for these highly individualistic participants, first information samples already strongly predicted prosocial choices in the DG even under low time pressure (at −1*SD* SVO *b_info1_* = 0.619, 95% CI = [0.251, 0.987], *z* = 3.294, post hoc two-tailed *p* < .001, *r* = 0.168).

Furthermore, comprehensive model comparison using stepwise regression that removed the highest-order predictor with the smallest *Z*-value until only first-order predictors were left identified the model that *only* included one two-way interaction between first information samples and time pressure as the most parsimonious (*BIC_min_* = 46125.082). Inclusion of additional two-way interactions between (1) SVO and game context, (2) SVO and time pressure, and (3) game context and time pressure further decreased the parsimony of the model without explaining significantly more variance (Model 1: ΔBIC = 0.622; Model 2: ΔBIC = 9.404; Model 3: ΔBIC = 10.160). Notably, these results suggest that after accounting for differences in information prioritization and subsequent search (see Supplementary Table S4), neither individual differences in SVO nor context further influenced changes in prosociality under time pressure independently. Moreover, differences in information search also seemed to largely account for why SVO was more predictive of prosocial choices in the DG compared to the UG.

## Discussion

While popular theories of social behavior have sought to explain variability in people’s selfishness and prosociality by appealing to dual-process dynamics of dispositional preferences (Böckler et al., 2016; Moore & Loewenstein, 2004; Rand, 2016; Rand et al., 2012; Yamagishi et al., 2017; Zaki & Mitchell, 2013), recent work highlights that they often fail to consider how different incentive structures across social interactions, as well as human constraints on information processing, shape the mechanistic expression of these preferences (Teoh et al., 2020; Teoh & Hutcherson, 2022; Thielmann et al., 2020). Here, we demonstrate that people navigate social interactions across different contexts by flexibly calibrating their information priorities to *balance* their own social preferences against the structural incentives and constraints of the social environment. Specifically, people increasingly prioritized both information that aligned with their dispositional social preference, and information that was incentive-compatible with the social environment under time pressure. For example, a selfishly predisposed person tended to further prioritize searching for self-relevant information under time constraints even in the Ultimatum game, where selfish behavior is disincentivized, but to a lesser degree than a similarly selfish person in the Dictator game, where there were no such disincentives. Consequently, information priorities in the Ultimatum game reflected an integration of both personal preferences and contextual incentives, tracking peoples’ dispositional preferences less reliably than information priorities in the Dictator game. Moreover, we found that accounting for these differences in information prioritization was sufficient to explain variability in how individuals adapted their prosocial choices across the game contexts and time constraints. In other words, our results here suggest that differences in an individual’s prosocial behavior across social interactions with different environmental incentives and constraints might *largely* be driven by what information they ultimately prioritize during social decision-making.

Yet, unlike past work, time pressure did not increase prosociality in our study, even for the most prosocial of our participants in contexts where selfishness could be penalized (Bouwmeester et al., 2017; Rand, 2016). Instead, our results join growing evidence suggesting that time pressure increases overall selfishness in some contexts (Capraro & Cococcioni, 2016; Krawczyk & Sylwestrzak, 2018; Teoh & Hutcherson, 2022; Teoh et al., 2020). How can we reconcile these apparent contradictions with the broader literature showing that time pressure sometimes increases prosociality? One possible explanation is that our sample of participants did not include any hyperaltruistic participants (Rhoads et al., 2021, 2023)—that is, people who prioritize others’ welfare *over* their own interests—who our theory predicts are most likely to prioritize other-relevant information and act more prosocially under time pressure. Instead, all our participants, even if prosocial, cared more about their own outcomes than others, and thus prioritized self-relevant information and acted more selfishly under constraints. While our sample could theoretically be systematically less altruistic than the overall population, such an explanation seems unlikely given that research suggests few individuals hold such extreme prosocial preferences (Henrich et al., 2005), and meta-analytic evidence points to relatively consistent increases in prosociality under time pressure in some contexts (Bouwmeester et al., 2017; Rand, 2016). Instead, closer examination of participants’ behavior in the Ultimatum game compared to the Dictator game points to the specific social incentives employed in the Ultimatum game as the likely explanation for why we fail to find reliable increases in prosociality. Specifically, we found that differences in prosociality between the Dictator and Ultimatum games were greatest for individuals who were highly selfish. In other words, penalties for selfishness effectively deterred selfishly predisposed participants from enacting those preferences, but its effects were less consistent for increasingly prosocial participants. In addition, these disincentives themselves were not sufficiently powerful to counteract the preferential prioritization of self-information under time pressure, leading to a net prioritization of self-relevant information in the Ultimatum game. In contrast, studies showing that time pressure increased overall prosociality often employed Public Good games where mutual benefits of prosociality are emphasized: cooperation jointly maximizes participants’ own outcomes and their partners’ (Bouwmeester et al., 2017; Rand, 2016). One might expect such incentives to operate both more powerfully but also more uniformly across individuals. Future work should investigate how the nature of incentives shapes people’s information search and decision-making under constraints. Separately, we emphasize here that our findings do not preclude the possibility of intuitive preferences. Alternatively, they identify a potential mechanism underlying intuitive processing, suggesting that dual-process theorists could advance our understanding of the distinction between intuition and deliberation by examining whether information prioritization is driven by automatic or controlled attention (Corbetta & Shulman, 2002).

Our results here also extend a growing body of work emphasizing people’s flexibility in calibrating their own preferences against features of the social context when faced with decisions to act selfishly or prosocially (Hu et al., 2023; Li et al., 2022; Teoh & Hutcherson, 2022; Thielmann et al., 2020, 2022). We demonstrate for the first time how both personal social preferences *and* structural incentives of the social context *jointly* determine the strategic value of different pieces of information and drive prioritization of attention during decision-making, especially under time constraints. These priorities then disproportionately influence the choices people ultimately make under time constraints when further information search is limited, leading to the extremification of choice patterns (Chen & Krajbich, 2018). Notably, complementing research on the temporal dynamics of information processing during decision-making (Amasino et al., 2019; Chen, Zheng, et al., 2024; Chen, Zhu, et al., 2024; Maier et al., 2020; Sullivan et al., 2015), our work explicitly identifies attentional prioritization as a key mechanism by which these temporal dynamics emerge, and why: attentional priorities express how people flexibly navigate social interactions where their own dispositional preferences may conflict with structural incentives. Critically, these findings not only provide a parsimonious resolution to ongoing debates about time pressure’s influence on social behavior but also make clear predictions both about *whether* time pressure will increase or decrease prosociality in a new context and *for whom*. Given intervention science’s increasing emphasis on the personalization of behavioral interventions (Halperin & Schori-Eyal, 2020; Kizilcec et al., 2020), our unified attentional framework may shed light on relevant efforts to facilitate social interactions in the real world, such as how different individuals might adapt to workplace cultures that differentially emphasize competition or collaboration (Drago & Turnbull, 1991), or how the balance between individual and collective responsibility in public health messaging might be tailored to reach specific audiences (Ceylan & Hayran, 2021). It suggests that greater success could be achieved by focusing more on attention than habits. To do so, however, future work would have to develop more sophisticated models that explain and predict how people deploy their attention in ecologically valid social interactions where social incentives are rarely made explicit, and the amount of relevant information far outstrips our information processing capacities. Such work would be instrumental in optimizing the efficacy of interventions that seek to facilitate social cohesion and improve social well-being.

## Supplemental Material

sj-docx-1-spp-10.1177_19485506251314071 – Supplemental material for Information Prioritization Underpins the Flexible Expression of Social Preferences Under Time Constraints Supplemental material, sj-docx-1-spp-10.1177_19485506251314071 for Information Prioritization Underpins the Flexible Expression of Social Preferences Under Time Constraints by Yi Yang Teoh, Hyuna Cho and Cendri A. Hutcherson in Social Psychological and Personality Science
