# Supplementary material for: Information Prioritization Underpins the Flexible Expression of Social Preferences Under Time Constraints
Source: Soc Psychol Personal Sci. 2025 Jan 28;16(7):815–26. doi: 10.1177/19485506251314071 (PMC12301522; doi:10.1177/19485506251314071)
Supplement: sj-docx-1-spp-10.1177_19485506251314071 – Supplemental material for Information Prioritization Underpins the Flexible Expression of Social Preferences Under Time Constraints [file sj-docx-1-spp-10.1177_19485506251314071.docx]

**Supplementary Materials for**

“Information prioritization underpins the flexible expression of social preferences under time constraints.”

**Authors:** Yi Yang Teoh^1a^, Hyuna Cho^a^, Cendri A. Hutcherson^a,b,^

^a^ Department of Psychology, University of Toronto, Toronto, Canada

^b^ Department of Marketing, Rotman School of Management, University of Toronto, Toronto, Canada

**Table S1: Effects of first information sample, game context, and time pressure on total number of information samples, completeness of information search, and initial sampling duration**

| **Predictors** | **Estimated *b***  **[95% CI]** | | |
| --- | --- | --- | --- |
|  | **Total Number of Information Samples** | **Proportion Incomplete Search** | **Initial Sampling Duration** |
| **Main Effects** |  |  |  |
| Intercept | 0.589***  [0.556, 0.622] | -2.693***  [-3.018, -2.367] | 5.621***  [5.583, 5.660] |
| Game | 0.063  [-0.002, 0.129] | -1.444***  [-2.088, -0.801] | -0.137***  [-0.215, -0.060] |
| Time | -0.459***  [-0.467, -0.450]^a^ | 4.200***  [4.076, 4.324]^h^ | -0.166***  [-0.173, -0.159]^o^ |
| Initial Information Sample | 0.034***  [0.022, 0.045] | -0.348***  [-0.472, -0.224] | -0.067***  [-0.078, -0.057] |
| SVO | 0.004  [-0.029, 0.037]] | -0.119  [-0.447, 0.208] | -0.015  [-0.054, 0.024] |
| Game × Time | 0.154***  [0.137, 0.171]^b^ | -0.567***  [-0.812, -0.322]^i^ | -0.076***  [-0.090, -0.062]^p^ |
| Game × Initial Information Sample | -0.160***  [-0.183, -0.137] | -0.268*  [-0.515, -0.021] | 0.026*  [0.005, 0.048] |
| Time × Initial Information Sample | 0.130***  [0.113, 0.147] | -1.274***  [-1.501, -1.047] | -0.157***  [-0.171, -0.143] |
| Game × SVO | 0.006  [-0.060, 0.072] | -0.353  [-1.006, 0.300] | -0.039  [-0.117, 0.039] |
| Time × SVO | 0.014**  [0.005, 0.023] | -0.526***  [-0.669, -0.383] | -0.061***  [-0.069, -0.053] |
| Initial Information Sample × SVO | -0.094***  [-0.105, -0.083] | 0.602***  [0.459, 0.746] | 0.106***  [0.093, 0.118] |
| Game × Time × Initial Information Sample | -0.147***  [-0.181, -0.113]^c^ | 0.704**  [0.251, 1.156]^j^ | 0.095***  [0.066, 0.124]^q^ |
| Game × Time × SVO | 0.012  [-0.006, 0.030] | 0.307*  [0.022, 0.592] | 0.014  [-0.001, 0.029] |
| Game × SVO × Initial Information Sample | -0.071***  [-0.093, -0.048] | -0.097  [-0.383, 0.189] | -0.052***  [-0.077, -0.028] |
| Time × SVO × Initial Information Sample | -0.073***  [-0.091, -0.055] | 0.351**  [0.086, 0.616] | 0.066***  [0.050, 0.081] |
| Game × Time × SVO × Initial Information Sample | -0.170***  [-0.207, -0.134] | 0.864**  [0.337, 1.391] | 0.154***  [0.123, 0.185] |
| **Simple Effects** |  |  |  |
| Time (Dictator game) | -0.536***  [-0.549, -0.523]^d^ | 4.483***  [4.305, 4.662]^k^ | -0.128***  [-0.138, -0.117]^r^ |
| Time (Ultimatum game) | -0.382***  [-0.393, -0.370]^e^ | 3.917***  [3.747, 4.086]^l^ | -0.204***  [-0.213, -0.195]^s^ |
| Game × Time (Sample $Other first) | 0.081***  [0.053, 0.108]^f^ | -0.215  [-0.596, 0.166]^m^ | -0.029*  [-0.052, -0.006]^t^ |
| Game × Time (Sample $Self first) | 0.227***  [0.207, 0.248]^g^ | -0.919***  [-1.196, -0.641]^n^ | -0.124***  [-0.140, -0.107]^u^ |

Note: Mixed-effects generalized poisson regression on total information sample count. Mixed-effects logistic regression on incomplete information search (complete = 0; incomplete = 1). Mixed-effects linear regression on log transformed initial sampling duration(ms). Trials with no recorded information samples were discarded. Game context (Ultimatum = 0.5; Dictator = −0.5), time pressure (High = 0.5, Low = −0.5) and initial information sample( $Self = −0.5, $Other = 0.5) were effect coded. SVO scores were mean-centered. Simple effects indicate the effect of the target variable(s) at the level of other variables specified in parentheses. Participants were treated as a random effect with varying intercepts.†p < .10, *p < .05, **p < .01, ***p < .001 (two-tailed)

^a^Pre-registered main hypothesis 1a: one-tailed p < .001

^b^Pre-registered main hypothesis 2b-i: one-tailed p < .001

^c^Pre-registered main hypothesis 5a-ii: one-tailed p < .001

^d^Pre-registered main hypothesis 1a: one-tailed p < .001

^e^Pre-registered main hypothesis 1a: one-tailed p < .001

^f^Pre-registered main hypothesis 5a-ii: one-tailed p = 1, two-tailed p < .001

^g^Pre-registered main hypothesis 5b-ii: one-tailed p < .001

^h^Pre-registered main hypothesis 1b: one-tailed p < .001

^i^Pre-registered main hypothesis 2b-ii: one-tailed p < .001

^j^Pre-registered main hypothesis 5a-iii: one-tailed p = .001

^k^Pre-registered main hypothesis 1b: one-tailed p < .001

^l^Pre-registered main hypothesis 1b: one-tailed p < .001

^m^Pre-registered main hypothesis 5a-iii: one-tailed p = 1, two-tailed p = .296

^n^Pre-registered main hypothesis 5b-iii: one-tailed p < .001

^o^Pre-registered main hypothesis 1c: one-tailed p < .001

^p^Pre-registered main hypothesis 2b-iii: one-tailed p < .001

^q^Pre-registered main hypothesis 5a-i: one-tailed p < .001

^r^Pre-registered main hypothesis 1c: one-tailed p < .001

^s^Pre-registered main hypothesis 1c: one-tailed p < .001

^t^Pre-registered main hypothesis 5a-i: one-tailed p = 1, two-tailed p = .015

^u^Pre-registered main hypothesis 5b-i: one-tailed p < .001

**Table S2: Effects of Social Value Orientation (SVO), game context, and time pressure on prosocial choices**

| **Predictors** | **Estimated *b***  **[95% CI]** |
| --- | --- |
| **Main Effects** |  |
| Intercept | -0.255***  [-0.368, -0.141] |
| Time | -0.272***  [-0.317, -0.227] |
| Game | 0.716***  [0.489, 0.944] |
| SVO | 0.567***  [0.452, 0.681] |
| Time × Game | 0.108*  [0.018, 0.197]^a^ |
| Time × SVO | 0.084***  [0.036, 0.132] |
| Game × SVO | -0.361**  [-0.590, -0.132] |
| Time × Game × SVO | 0.030  [-0.066, 0.126]^b^ |
| **Simple Effects** |  |
| Time (Dictator game at mean SVO) | -0.326***  [-0.392, -0.260]^c^ |
| Time (Ultimatum game at mean SVO) | -0.218***  [-0.279, -0.158]^d^ |
| SVO (Dictator game under low time pressure) | 0.713***  [0.544, 0.881]^e^ |
| SVO (Ultimatum game under low time pressure) | 0.336***  [0.175, 0.497]^f^ |
| SVO (Dictator game under high time pressure) | 0.782***  [0.611, 0.952] |
| SVO (Ultimatum game under high time pressure) | 0.436***  [0.273, 0.598] |
| Game × SVO (Low time pressure) | -0.377**  [-0.610, -0.144]^g^ |
| Game × SVO (High time pressure) | -0.346**  [-0.581, -0.111] |
| Time × SVO (Dictator game) | 0.069†  [-0.005, 0.143]^h^ |
| Time × SVO (Ultimatum game) | 0.099**  [0.038, 0.161]^i^ |

Note: The table shows unstandardized coefficients from a mixed-effects logistic regression on prosociality (selfish = 0, prosocial = 1). Game context (ultimatum = 0.5, dictator = −0.5) and time pressure (high = 0.5, low = −0.5) were effects coded. SVO scores were mean-centered. Simple effects indicate the effect of the target variable at the level of other variables specified in parentheses. Participants were treated as a random effect with varying intercepts. Values in parentheses are 95% confidence intervals. †p = .066, *p < .05, **p < .01, ***p < .001 (two-tailed)

^a^Pre-registered main hypothesis 4a: one-tailed p = .009

^b^Pre-registered exploratory hypothesis 4: one-tailed p = 1, two-tailed p = .535

^c^Pre-registered main hypothesis 4b: one-tailed p < .001

^d^Pre-registered main hypothesis 4c: one-tailed p < .001

^e^Pre-registered exploratory hypothesis 1: one-tailed p < .001

^f^Pre-registered exploratory hypothesis 1: one-tailed p < .001

^g^Pre-registered exploratory hypothesis 2: one-tailed p < .001

^h^Pre-registered exploratory hypothesis 3: one-tailed p = .033

^i^Pre-registered exploratory hypothesis 3: one-tailed p < .001

**Table S3: Effects of Social Value Orientation (SVO), game context, and time pressure on information priorities**

| **Predictors** | **Estimated *b***  **[95% CI]** |
| --- | --- |
| **Main Effects** |  |
| Intercept | -1.417***  [-1.889, -0.944]^a^ |
| Time | -0.374***  [-0.442, -0.306] |
| Game | 1.826***  [0.908, 2.744]^b^ |
| SVO | 0.965***  [0.488, 1.443] |
| Time × Game | 0.305***  [0.169, 0.441]^c^ |
| Time × SVO | 0.657***  [0.576, 0.737]^d^ |
| Game × SVO | -1.222**  [-2.140, -0.304] |
| Time × Game × SVO | 0.131  [-0.029, 0.292]^e^ |
| **Simple Effects** |  |
| Time (Dictator game at mean SVO) | -0.526***  [-0.629, -0.423]^f^ |
| Time (Ultimatum game at mean SVO) | -0.221***  [-0.310, -0.132]^g^ |
| Time × SVO (Dictator game) | 0.591***  [0.456, 0.726]^h^ |
| Time × SVO (Ultimatum game) | 0.722***  [0.635, 0.809]^i^ |
| SVO (Dictator game under low time pressure) | 1.281***  [0.593, 1.969] |
| SVO (Ultimatum game under low time pressure) | -0.007  [-0.661, 0.648] |
| SVO (Dictator game under high time pressure) | 1.872***  [1.180, 2.563] |
| SVO (Ultimatum game under high time pressure) | 0.716*  [0.053, 1.379] |
| Game × SVO (Low time pressure) | -1.287**  [-2.228, -0.346] |
| Game × SVO (High time pressure) | -1.156*  [-2.088, -0.224] |

Note: The table shows unstandardized coefficients from a mixed-effects logistic regression on initial information sample($Self = 0; $Other = 1). Trials with no recorded information samples were discarded. Game context (ultimatum = 0.5, dictator = −0.5) and time pressure (high = 0.5, low = −0.5) were effects coded. SVO scores were mean-centered. Simple effects indicate the effect of the target variable at the level of other variables specified in parentheses. Participants were treated as a random effect with varying intercepts. Values in parentheses are 95% confidence intervals. †p < .10, *p < .05, **p < .01, ***p < .001 (two-tailed)

^a^Pre-registered main hypothesis 2a-i: one-tailed p < .001

^b^Pre-registered main hypothesis 2a-ii: one-tailed p < .001

^c^Pre-registered main hypothesis 2a-iii: one-tailed p < .001

^d^Pre-registered exploratory hypothesis 3: one-tailed p < .001

^e^Pre-registered exploratory hypothesis 4: one-tailed p = 1, two-tailed p = .109

^f^Pre-registered main hypothesis 2a-iii: one-tailed p < .001

^g^Pre-registered main hypothesis 2a-iii: one-tailed p = 1, two-tailed p < .001

^h^Pre-registered exploratory hypothesis 3: one-tailed p < .001

^i^Pre-registered exploratory hypothesis 3: one-tailed p < .001

**Table S4: Effects of first information sample, time pressure, Social Value Orientation (SVO), and game context on trial-level prosocial choice.**

| **Predictors** | **Estimated *b***  **[95% CI]** | | | | |
| --- | --- | --- | --- | --- | --- |
|  | **Full Model** | **Selected Model** | **Model 1** | **Model 2** | **Model 3** |
| **Main Effects** |  |  |  |  |  |
| Intercept | -0.194***  [-0.303, -0.085] | -0.225***  [-0.336, -0.114] | -0.207***  [-0.316, -0.099] | -0.227***  [-0.338, -0.116] | -0.226***  [-0.337, -0.115] |
| Game | 0.653***  [0.435, 0.871] | 0.651***  [0.428, 0.873] | 0.652***  [0.435, 0.869] | 0.651***  [0.429, 0.874] | 0.652***  [0.430, 0.874] |
| Time | -0.189***  [-0.242, -0.137] | -0.195***  [-0.241, -0.149] | -0.195***  [-0.241, -0.149] | -0.200***  [-0.246, -0.153] | -0.197***  [-0.243, -0.151] |
| SVO | 0.516***  [0.404, 0.627] | 0.520***  [0.408, 0.632] | 0.526***  [0.417, 0.636] | 0.522***  [0.410, 0.634] | 0.520***  [0.408, 0.632] |
| Initial information sample | 0.452***  [0.344, 0.560] | 0.475***  [0.372, 0.577] | 0.475***  [0.373, 0.578] | 0.466***  [0.363, 0.569] | 0.473***  [0.370, 0.575] |
| Remainder sampling bias | 0.258***  [0.135, 0.382] | 0.283***  [0.163, 0.403] | 0.285***  [0.166, 0.405] | 0.274***  [0.154, 0.395] | 0.280***  [0.160, 0.400] |
| Initial information sample × Time | 0.517***  [0.323, 0.711]^a^ | 0.354***  [0.258, 0.451] | 0.355***  [0.259, 0.452] | 0.347***  [0.250, 0.445] | 0.349***  [0.252, 0.447] |
| Game × SVO | -0.361**  [-0.584, -0.138] |  | -0.355**  [-0.573, -0.137] |  |  |
| Time × SVO | 0.019  [-0.041, 0.080] |  |  | 0.027  [-0.022, 0.075] |  |
| Game × Time | 0.053  [-0.051, 0.158] |  |  |  | 0.029  [-0.062, 0.120] |
| Initial information sample × Game | -0.222*  [-0.438, -0.006] |  |  |  |  |
| Initial information sample × SVO | -0.108  [-0.226, 0.010] |  |  |  |  |
| Remainder sampling bias × Game | -0.109  [-0.356, 0.138] |  |  |  |  |
| Remainder sampling bias × Time | 0.215  [-0.012, 0.441] |  |  |  |  |
| Remainder sampling bias × SVO | -0.127  [-0.259, 0.006] |  |  |  |  |
| Game × Time × SVO | -0.044  [-0.164, 0.076] |  |  |  |  |
| Initial information sample × Game × Time | 0.299  [-0.089, 0.687] |  |  |  |  |

| **Predictors** | **Estimated *b***  **[95% CI]** | | | | |
| --- | --- | --- | --- | --- | --- |
|  | **Full Model** | **Selected Model** | **Model 1** | **Model 2** | **Model 3** |
| **Main Effects** |  |  |  |  |  |
| Initial information sample × Game × SVO | -0.101x  [-0.337, 0.136] |  |  |  |  |
| Initial information sample × Time × SVO | 0.108  [-0.101, 0.316] |  |  |  |  |
| Remainder sampling bias × Time × SVO | 0.100  [-0.143, 0.344] |  |  |  |  |
| Initial information sample × Game × Time × SVO | -0.512*  [-0.927, -0.097] |  |  |  |  |
| Remainder sampling bias × Game × Time × SVO | -0.572*  [-1.058, -0.087] |  |  |  |  |
| **Simple Effects** |  |  |  |  |  |
| Initial information sample × Time (Dictator Game at mean SVO) | 0.367**  [0.099, 0.636]^b^ |  |  |  |  |
| Initial information sample × Time (Ultimatum Game at mean SVO) | 0.666***  [0.389, 0.943]^c^ |  |  |  |  |
| Initial information sample (High time pressure in Dictator game at mean SVO) | 0.747***  [0.560, 0.934]^d^ |  |  |  |  |
| Initial information sample (High time pressure in Ultimatum game at mean SVO) | 0.674***  [0.484, 0.865]^e^ |  |  |  |  |
| **BIC** | 46265.024 | 46125.082^f^ | 46125.703 | 46134.486^f^ | 46135.242 |

Note: Mixed−effects logistic regression on prosocial choice on each trial (Selfish = 0; Prosocial = 1). Trials with no recorded information samples were discarded. Game context (Ultimatum = 0.5; Dictator = −0.5), time pressure (High = 0.5, Low = −0.5) and initial information sample ($Other = 0.5, $Self = −0.5) were effect−coded. SVO scores were mean-centered. We controlled for effects of remainder sampling biases, calculated by subtracting 0.5 from the proportion of samples of $Other over samples of $Self and $Other, not including the initial information sample (Only looked at $Self = −0.5; Only looked at $Other = 0.5). Simple effects indicate the effect of the target variable(s) at the level of other variables specified in parentheses. Participants were treated as a random effect with varying intercepts. †p < .10, *p < .05, **p < .01, ***p < .001 (two-tailed)

^a^Pre-registered hypothesis 3a: one-tailed p < .001

^b^Pre-registered hypothesis 3a: one-tailed p = .004

^c^Pre-registered hypothesis 3a: one-tailed p < .001

^d^Pre-registered hypothesis 3a: one-tailed p < .001

^e^Pre-registered hypothesis 3a: one-tailed p < .001

^f^Pre-registered hypothesis 3b: ΔBIC(Selected Model-Model 2) < -5
